# Associations of social avoidance and sport engagement with physical health indicators among schoolchildren: a cross-sectional study

**DOI:** 10.3389/fpubh.2026.1890401

**Published:** 2026-09-21

**Authors:** Yameng Li

**Affiliations:** Department of Physical Education, Northwestern Polytechnical University, Xi’an, China

**Keywords:** social avoidance, sport engagement, sport participation, physical fitness, body mass index, overweight and obesity, school-aged children

## Abstract

**Background:**

This study examined cross-sectional associations of social avoidance with sport engagement and of sport engagement with physical health indicators among schoolchildren. Potential nonlinear associations were also assessed. Exploratory latent profile analysis (LPA) examined whether cognitive, affective, and relational engagement provided information beyond the continuous total engagement score.

**Methods:**

This cross-sectional study included 923 students in Grades 3–5 from one Beijing school. Questionnaires assessed social avoidance and sport engagement across sport settings. Routine school records provided physical fitness, height, and weight, from which a BMI-excluded physical fitness score was calculated. Classroom-clustered linear and logistic regression models examined associations of social avoidance with total sport engagement and of sport engagement with BMI-excluded physical fitness, BMI, and overweight/obesity. Models adjusted for sex, grade, school-based sport participation, and out-of-school sport participation. Holm correction was applied to four focal associations and four corresponding quadratic tests. LPA was exploratory and accounted for classroom clustering.

**Results:**

The adjusted regression analyses included 913 students. Higher social avoidance was associated with lower total sport engagement (*β* = −0.106, 95% CI [−0.178, −0.034]). Higher sport engagement was associated with higher BMI-excluded physical fitness (*β* = 0.189, 95% CI [0.118, 0.260]), lower BMI (*β* = −0.141, 95% CI [−0.212, −0.070]), and lower odds of overweight/obesity (OR = 0.721, 95% CI [0.628, 0.827]). All four associations remained significant after Holm correction, whereas none of the quadratic terms did. LPA criteria did not identify an unequivocally optimal solution. A descriptive three-class representation yielded model-estimated proportions of 11.0, 39.7, and 49.3% for low, moderate, and high engagement levels, respectively. The three dimensions increased broadly in parallel, and assigned class membership was not used in outcome analyses.

**Conclusion:**

Higher social avoidance co-occurred with lower sport engagement, whereas higher sport engagement co-occurred with higher BMI-excluded physical fitness, lower BMI, and lower odds of overweight/obesity. These associations were small to modest and do not establish temporal or causal relationships. The exploratory classes primarily represented ordered levels along a general engagement continuum and did not support profile-based risk classification.

## 1 Introduction

Insufficient physical activity and excessive sedentary behavior are major public health concerns among school-aged children. The World Health Organization recommends that children and adolescents engage in an average of at least 60 min of moderate-to-vigorous physical activity per day and limit sedentary behavior, especially recreational screen time (1). Regular physical activity is associated with cardiorespiratory health, musculoskeletal health, weight status, body composition, mental health, and cognitive and academic outcomes in young people (2–4). In contrast, sedentary behavior and excessive screen time are associated with unfavorable body composition, lower fitness, cardiometabolic risk, and poorer psychosocial health (5). Health-related physical fitness (HRF) and body mass index (BMI) are two important indicators of children’s physical health. HRF is widely regarded as an important marker of health in children and adolescents and has predictive value for later cardiometabolic health, body composition, and skeletal health (6, 7). Compared with skill-related fitness, HRF is more directly related to healthy growth, disease prevention, and long-term health risk reduction (8). BMI does not directly measure body fat, but it remains a practical indicator of weight status and overweight or obesity risk. It is also related to cardiometabolic risk, physical activity level, and fitness performance in youth (9, 10). Thus, HRF and BMI provide complementary information about children’s functional health and weight-related risk.

Schools are key settings for promoting children’s physical activity. School-based physical activity programs can improve physical activity and fitness among children and adolescents (11). Physical education is also associated with improvements in HRF and fundamental motor skills in youth (12). Beyond physical education, organized sport and extracurricular sport activities provide opportunities for children to be active, develop motor skills, build social competence, and develop active lifestyles (13). Participation in youth sport has also been associated with physical and psychosocial benefits (14). Evidence also links weight status and sport-related activity with physical fitness in school-aged children (15). Therefore, school physical education, extracurricular sport, and organized sport are important contexts for understanding differences in children’s HRF and BMI.

However, opportunities for physical activity and activity dose do not fully describe children’s experiences in sport. The benefits associated with youth sport may also vary with program organization, instructional quality, and the quality of participation experiences (16). Enjoyment is positively associated with physical activity and fitness, and physical activity may partly account for the association between enjoyment and fitness (17). Self-determination theory identifies enjoyment, autonomous motivation, and basic psychological need satisfaction as important for sustained participation (18). Intrinsic motivation in physical education has also been associated with objectively measured daily physical activity among adolescents (19). Research on extracurricular physical activity similarly highlights the relevance of enjoyment, motivation, and BMI to developmental outcomes (20). This evidence supports examining not only whether and how much children participate, but also their psychological involvement in sport.

Sport engagement is a multidimensional indicator of children’s psychological involvement in sport (21). Unlike participation frequency, duration, or activity dose, it captures cognitive, affective, and relational engagement—that is, how children attend to, feel about, and connect with others during sport activities (21, 22). Because sport can make motor performance and body characteristics visible to peers, motivational experiences, peer climate, and body-related concerns may shape children’s participation experiences (23–27). Sport engagement may therefore provide information about children’s experiences in sport that is not captured by participation setting or activity dose alone and may be associated with physical fitness and weight-related indicators.

In this context, social avoidance may be relevant to children’s sport engagement. As a form of social withdrawal, it is characterized by avoiding social interaction or withdrawing from social situations (28). Unlike peer conflict, bullying, or rejection, social avoidance may be less visible at school, although socially avoidant children may be less interactive and less involved in group activities (28, 29). Physical education and sport commonly involve peer interaction, group practice, cooperation, competition, and social comparison (25, 27). These features may be challenging for socially avoidant children and may be associated with lower cognitive, affective, and relational engagement. Consistent with this possibility, shy or socially withdrawn children report lower sport involvement and may be less likely to participate in team-based or evaluative sport settings (29–31).

Previous research on social withdrawal and sport has focused largely on participation and internalizing problems (21, 29, 32–34). However, relatively little is known about how multidimensional psychological engagement in sport relates to social avoidance and physical health characteristics among younger schoolchildren, particularly after accounting separately for school-based and out-of-school sport participation. Weight status may also be relevant because overweight and obesity have been associated with bullying and negative peer interactions (35, 36), while higher BMI has been associated with lower physical activity (37). Examining social avoidance in relation to sport engagement, and sport engagement in relation to the BMI-excluded physical fitness score, BMI, and overweight/obesity status, may clarify how interpersonal, psychological, and physical health characteristics co-occur among schoolchildren.

Although the total score provides a concise measure of overall sport engagement, it may obscure uneven patterns across the cognitive, affective, and relational dimensions. Latent profile analysis (LPA) offers an exploratory person-centered approach for examining whether such patterns exist. It has been used to identify physical literacy profiles and compare physical activity across profiles in child movement research (38), as well as to examine physical activity, sedentary behavior, and broader health behavior patterns among children and adolescents (39, 40). Nevertheless, LPA may divide a continuous distribution into ordered levels rather than identify qualitatively distinct groups, particularly when indicators are bounded or show ceiling effects. Its value therefore depends on model stability, classification quality, substantive interpretability, and whether it provides information beyond the total score.

Therefore, this cross-sectional study examined whether social avoidance was associated with the total sport engagement score and whether the total sport engagement score was associated with the BMI-excluded physical fitness score, BMI, and overweight/obesity status among Chinese schoolchildren. These associations were examined after accounting for school-based and out-of-school sport participation. Potential nonlinearity was assessed for each association. Exploratory LPA was used to determine whether the cognitive, affective, and relational dimensions provided person-centered information beyond the total engagement score. Three research questions were addressed: (1) Is social avoidance associated with the total sport engagement score, and is this association nonlinear? (2) Is the total sport engagement score associated with the BMI-excluded physical fitness score, BMI, and overweight/obesity status, and are these associations nonlinear? (3) Does exploratory LPA reveal dimension-specific configurations, or does it primarily reproduce ordered levels of overall sport engagement?

## 2 Materials and methods

### 2.1 Study design and participants

This cross-sectional study was conducted from November 25 to December 8, 2025, in one public school in Beijing, China. Students in Grades 3–5 were invited to participate. Among the 923 students included in the analytical sample, 751 had valid birthdate records. Their mean age at the end of data collection was 9.71 ± 0.89 years (range = 7.35–12.21 years). Questionnaire data were collected in classrooms by trained researchers with assistance from classroom teachers. Health-related physical fitness data were obtained from routine school fitness testing conducted during the same semester. Questionnaire responses and fitness records were matched using student identification codes.

The participating school was an urban public school located in the Beijing Economic-Technological Development Area. It followed the national compulsory education curriculum and implemented the Chinese National Student Physical Fitness Standard. The findings should therefore be interpreted as evidence from one urban public-school setting rather than as population-level estimates for children in Beijing or China.

Grades 3–5 were included because peer interaction, social comparison, and group participation become increasingly salient during late childhood, when social withdrawal may have clearer adjustment implications (41, 42). In addition, primary-school physical fitness testing in China is organized and interpreted primarily by grade rather than chronological age. Grade was therefore used as the principal indicator of school stage (43).

The original dataset contained 1,110 records. Sequential screening excluded 187 records (16.8%), resulting in an analytical sample of 923 students. Exclusions were due to missing sex or grade information (*n* = 13), missing social avoidance item(s) (*n* = 20), missing sport engagement item(s) (*n* = 56), sport engagement responses outside the permitted 1–4 range (*n* = 11), missing data required to calculate the BMI-excluded physical fitness score or BMI (*n* = 50), and unverified classroom identifiers (*n* = 37). Because the criteria were applied sequentially, these counts were mutually exclusive. Complete item-level data were required to calculate the social avoidance and sport engagement scores.

Ten additional students had missing data on one or both sport-participation covariates. They were retained in the descriptive and measurement analyses and the exploratory latent profile analysis but excluded from the fully adjusted association models, which included 913 students. To evaluate potential selection bias, included and excluded students were compared using all available data. Pearson’s chi-square tests were used for sex and grade, Welch’s independent-samples *t* tests for continuous variables, and Cohen’s *d* for standardized mean differences. Screening procedures, variable-level missingness, and comparisons between included and excluded students are reported in Supplementary Table S1.

The study was approved by the Medical Ethics Committee of Northwestern Polytechnical University (approval no. 202502091) and conducted in accordance with the Declaration of Helsinki. Written informed consent was obtained from parents or legal guardians, and child assent was obtained before data collection.

### 2.2 Study size and statistical considerations

The study size was determined by the number of participating students who provided eligible data during the study period rather than by a power calculation tailored to a single hypothesis. After sequential screening, the descriptive and measurement analyses and the exploratory latent profile analysis included 923 students. The fully adjusted regression models included 913 students nested within 29 classrooms. In the overweight/obesity model, 195 outcome events were observed, and seven coefficients, including the intercept, were estimated, corresponding to approximately 28 events per coefficient. Statistical adequacy was evaluated with reference to the number of classrooms, the number of outcome events, confidence-interval precision, model convergence, and model stability. Because the number of classrooms was modest, the primary regression models used classroom-clustered CR1 standard errors with a finite-sample correction and a t reference distribution with 28 degrees of freedom. The latent profile analysis was treated as exploratory, and its adequacy was assessed using model convergence, log-likelihood replication, classification diagnostics, and the substantive interpretability of the candidate solutions.

### 2.3 Measures

#### 2.3.1 Social avoidance

Social avoidance was assessed using the four-item Social Avoidance Scale, which was grounded in the social-motivational conceptualization of avoidant withdrawal (44) and subsequently developed and validated for Chinese elementary- and middle-school students (45). Items describe active avoidance of peer interaction and are rated from 1 (“never”) to 5 (“always”). The four items were summed, with higher scores indicating greater social avoidance. An example item is “I do not want to play with other children”.

The original validation sample included elementary-school children with a mean age of 10.25 years, which was close to the mean age of 9.71 years among participants with valid birthdate records in the present analytical sample. Trained researchers administered the questionnaires in classrooms with assistance from classroom teachers. Standardized instructions and response options were explained, and children could ask for clarification. No formal cognitive interviews were conducted.

Internal consistency was acceptable (Cronbach’s *α* = 0.794). Because the items were ordinal, a one-factor confirmatory factor analysis (CFA) was estimated using the weighted least squares mean- and variance-adjusted estimator (WLSMV), with classroom clustering taken into account. Model fit was good, *χ*^2^(2) = 2.355, *p* = 0.308, CFI = 1.000, TLI = 0.999, RMSEA = 0.014 (90% CI [0.000, 0.068]), and SRMR = 0.006. Standardized factor loadings ranged from 0.736 to 0.826 (all *p* < 0.001).

#### 2.3.2 Sport engagement

Sport engagement was assessed using the nine-item Chinese sport-context version of the Snapshot Survey of Engagement Tool–Revised (21, 22, 46–48). The Chinese version was developed with permission from the original authors through translation and back-translation, sport-specific wording adaptation, and expert review. It was previously evaluated in 535 Chinese students in Grades 3–7, supporting its three-factor structure and measurement invariance across elementary- and middle-school students. No further item-level adaptation was made in the present study.

The measure comprises three cognitive, three affective, and three relational engagement items, rated from 1 (“never”) to 4 (“often”). Example items include “I really focus on sport activities when I am doing them,” “I enjoy sport activities and have fun when I am involved,” and “Sport activities connect me with other people.” Subscale scores were calculated by averaging the corresponding three items, and the nine items were summed to obtain an overall score ranging from 9 to 36. Higher scores indicate greater engagement. The instructions referred to sport activities generally rather than a specific setting; responses could therefore reflect physical education, other school-based sport activities, or out-of-school sport. Participation in school-based and out-of-school sport was assessed separately, as described in Section 2.3.3.

Internal consistency was acceptable for the cognitive (*α* = 0.744), affective (*α* = 0.711), and relational (*α* = 0.772) subscales and good for the overall scale (*α* = 0.889). A three-factor CFA using WLSMV treated the items as ordered categorical indicators and showed acceptable fit, *χ*^2^(24) = 107.582, *p* < 0.001, CFI = 0.991, TLI = 0.986, RMSEA = 0.061, 90% CI [0.050, 0.073], and SRMR = 0.021. Standardized loadings ranged from 0.618 to 0.863 (all *p* < 0.001). Factor correlations were high (*r* = 0.908–0.983), indicating limited separation among the dimensions. The overall score was therefore used in the primary continuous analyses, while the subscale scores were retained for the exploratory person-centered analyses.

For comparison, a one-factor model also showed acceptable but descriptively poorer fit, *χ*^2^(27) = 150.145, *p* < 0.001, CFI = 0.986, TLI = 0.982, RMSEA = 0.070 (90% CI [0.060, 0.081]), and SRMR = 0.025.

#### 2.3.3 School-based and out-of-school sport participation

The questionnaire separately assessed participation in school-based and out-of-school sport. School-based participation referred to sport clubs or enrichment courses organized by the school, whereas out-of-school participation referred to organized sport programs attended outside school. Each variable was coded as 0 = no and 1 = yes and was included as a covariate. Additional information was collected on sport type, weekly frequency, session duration, and years of participation but was not included in the primary adjusted models. The two binary variables captured participation setting, whereas the sport engagement scale assessed children’s psychological engagement in sport activities.

#### 2.3.4 Health-related physical fitness, BMI, and weight status

Health-related physical fitness, height, and weight data were obtained from routine school fitness testing conducted during the same autumn semester and within a similar time window as the questionnaire survey, which was administered from November 25 to December 8, 2025. Testing followed the Chinese National Student Physical Fitness Standard and was administered by trained school physical education teachers using the standardized instruments required for school fitness testing. No additional research-specific standardized warm-up was introduced. Each test result was recorded once, without repeat trials or selection of the best performance.

The fitness assessments comprised vital capacity, the 50-m sprint, sit-and-reach, 1-min rope skipping, and 1-min sit-ups. Grade 5 students additionally completed the 50-m × 8 shuttle run. To remove the BMI component from the composite fitness score, the BMI-excluded physical fitness score was calculated as [(base fitness score − 0.15 × BMI standard score)/0.85] + bonus points, where the base fitness score referred to the weighted 100-point composite before bonus points. Bonus points of up to 20 were retained in accordance with the national standard. Higher scores indicated better physical fitness (43).

BMI was calculated from measured height and weight using the formula: BMI = weight (kg)/[height (m)]^2^. Height and weight were measured using the standardized student fitness-testing instruments used by the school. BMI was analyzed as a continuous variable. Weight status was classified as underweight, normal weight, overweight, or obese using the sex- and grade-specific BMI thresholds of the Chinese National Student Physical Fitness Standard (2014 Revision) (43). The thresholds applied to students in Grades 3–5 are provided in Supplementary Table S2. For the binary analysis, overweight and obesity were combined and coded as 1, whereas underweight and normal weight were combined and coded as 0. No additional fitness or anthropometric testing was conducted specifically for this study.

### 2.4 Data analysis

Analyses were conducted using SPSS 26.0, Mplus 9.1, and Python 3.12. All tests were two-sided, with *α* = 0.05. Continuous variables are reported as means and standard deviations and categorical variables as frequencies and percentages. Sport engagement distributions were additionally described using medians, interquartile ranges, skewness, kurtosis, and maximum-score proportions (Supplementary Table S3). Internal consistency was assessed using Cronbach’s *α*. CFA models were estimated using WLSMV, with questionnaire items treated as ordered categorical indicators.

Variable-level missingness was examined before analysis. Included and excluded students were compared using Welch’s t tests for continuous variables and Pearson’s chi-square tests for categorical variables, with Cohen’s *d* and Cramér’s *V* reported as effect-size measures. Pearson correlations were used to describe bivariate associations.

The primary adjusted analyses included 913 students. The total sport engagement score was regressed on social avoidance, whereas BMI-excluded physical fitness and BMI were separately regressed on the total sport engagement score. Binary logistic regression examined the association between the total sport engagement score and overweight/obesity. All models adjusted for sex, grade, school-based sport participation, and out-of-school sport participation. Boys, Grade 3, and nonparticipation in the corresponding sport setting were the reference categories. Continuous focal variables were standardized before analysis. Classroom-clustered CR1 standard errors with a finite-sample correction and a *t* (28) reference distribution were used to account for students nested within 29 classrooms. Intraclass correlation coefficients were estimated using random-intercept models with restricted maximum likelihood, and corresponding design effects are reported in Supplementary Table S4. Multicollinearity, influential observations, linear-model residuals, and logistic-model convergence were also examined.

Potential nonlinearity was assessed by adding a squared standardized focal predictor to each primary model. Holm correction was applied separately to the four prespecified focal associations and the four nonlinearity tests. Standardized regression coefficients, odds ratios, 95% confidence intervals, and model-level effect measures are reported. Quadratic effects are presented as unstandardized coefficients. Secondary classroom-clustered models examined associations between the total sport engagement score and the six individual physical fitness components. These models adjusted for the same covariates as the primary analyses, and Holm correction was applied across the six tests. The 50-m × 8 shuttle-run analysis was restricted to Grade 5 students and adjusted for sex, school-based sport participation, and out-of-school sport participation (Supplementary Table S9).

Latent profile analysis was conducted as an exploratory analysis of whether the cognitive, affective, and relational dimensions provided information beyond the total sport engagement score (49, 50). The three subscale means were treated as continuous indicators after their bounded distributions and maximum-score proportions had been examined (Supplementary Table S3). Two- through six-class models were estimated using robust maximum likelihood and TYPE = MIXTURE COMPLEX, with classroom specified as the cluster variable, equal within-class variances, and within-class covariances fixed at zero. As a sensitivity analysis, a two-class model allowing class-specific within-class variances was also estimated. The two-class model used 1,000 initial-stage starts and 200 final-stage optimizations; the three- through six-class models used 2,000 initial-stage starts and 400 final-stage optimizations. Model selection considered information criteria, entropy, estimated and modal class sizes, average posterior probabilities, classification matrices, log-likelihood replication, convergence, indicator distributions, and substantive interpretability. VLMR-LRT, adjusted LMR-LRT, and BLRT results were obtained from otherwise equivalent non-complex models because these tests were unavailable under the classroom-complex specification. Complete enumeration and classification diagnostics are reported in Supplementary Tables S5–S8. Most likely class membership was not used as an observed predictor or grouping variable in the outcome analyses; associations with social avoidance and physical health indicators were examined using the total sport engagement score.

## 3 Results

### 3.1 Sample characteristics and preliminary analyses

Comparisons between included and excluded students based on available data are presented in Supplementary Table S1. No statistically significant differences were found in sex (*p* = 0.347), grade (*p* = 0.191), total sport engagement (*p* = 0.340), BMI-excluded physical fitness (*p* = 0.094), or BMI (*p* = 0.697). Compared with excluded students, included students had slightly lower social avoidance scores (*p* = 0.030, *d* = −0.220). The observed differences were small, although potential selection bias cannot be completely excluded.

The final study sample comprised 923 students in Grades 3–5, including 372 students in Grade 3, 288 in Grade 4, and 263 in Grade 5. The sample included 478 boys and 445 girls. According to the school fitness records, 50 students were classified as underweight, 675 as normal weight, 114 as overweight, and 84 as obese.

Among students with available participation data, 389 of 921 (42.2%) participated in school-based sport clubs or enrichment courses, and 506 of 915 (55.3%) participated in out-of-school sport programs.

Descriptive statistics and Pearson correlations are presented in Table 1. The mean scores were 5.61 ± 2.41 for social avoidance, 27.35 ± 6.54 for total sport engagement, 101.29 ± 12.10 for BMI-excluded physical fitness, and 17.62 ± 3.24 for BMI. Social avoidance was negatively correlated with sport engagement (*r* = −0.120, *p* < 0.001) and BMI-excluded physical fitness (*r* = −0.081, *p* = 0.014), but was not significantly correlated with BMI (*r* = −0.024, *p* = 0.468). Sport engagement was positively correlated with BMI-excluded physical fitness (*r* = 0.247, *p* < 0.001) and negatively correlated with BMI (*r* = −0.123, *p* < 0.001). BMI-excluded physical fitness was negatively correlated with BMI (*r* = −0.293, *p* < 0.001).

**Table 1:** Descriptive statistics and correlations among the main variables.

Variable	M ± SD	1	2	3
1. Social avoidance	5.61 ± 2.41	—		
2. Sport engagement	27.35 ± 6.54	−0.120***	—	
BMI-excluded physical fitness	101.29 ± 12.10	−0.081*	0.247***	—
4. BMI	17.62 ± 3.24	−0.024	−0.123***	−0.293***

*N* = 923. Values are Pearson correlation coefficients. *p < 0.05, **p < 0.01, ***p < 0.001.

### 3.2 Sport engagement distributions and classroom clustering

The mean subscale scores were 3.05 ± 0.78 for cognitive engagement, 3.07 ± 0.80 for affective engagement, and 3.00 ± 0.85 for relational engagement. The maximum total sport engagement score was observed in 105 students (11.4%). The proportions attaining the maximum cognitive, affective, and relational subscale scores were 20.7, 24.3, and 23.2%, respectively, and 144 students (15.6%) attained the maximum score on both the affective and relational subscales. Subscale skewness ranged from −0.668 to −0.600. Across the nine items, the proportion selecting the maximum response ranged from 29.7 to 58.9% (Supplementary Table S3).

The 923 students were nested within 29 classrooms, with a mean classroom size of 31.83 students (range = 9–41). Intraclass correlation coefficients estimated from unconditional random-intercept models were 0.011 for social avoidance, 0.023 for total sport engagement, 0.023 for cognitive engagement, 0.016 for affective engagement, 0.020 for relational engagement, 0.289 for BMI-excluded physical fitness, and 0.050 for BMI. The corresponding design effects were 1.35, 1.72, 1.71, 1.50, 1.63, 9.90, and 2.53, respectively. After adjustment for grade, the residual ICCs were 0.101 for BMI-excluded physical fitness and 0.019 for BMI. Classroom-clustered standard errors with a small-sample correction were used in all subsequent regression models.

### 3.3 Association between social avoidance and sport engagement

The adjusted analysis included 913 students with complete covariate data. After adjustment for sex, grade, school-based sport participation, and out-of-school sport participation, higher social avoidance was associated with a lower total sport engagement score (*β* = −0.106, 95% CI [−0.178, −0.034], *p* = 0.005; Holm-adjusted *p* = 0.005). The adjusted R^2^ for the model was 0.082. Complete model coefficients are presented in Table 2.

**Table 2:** Classroom-clustered, covariate-adjusted associations among social avoidance, the total sport engagement score, and physical health indicators.

Outcome	Predictor	Estimate (*β* or OR)	95% CI	*p*	Holm-adjusted *p*
Sport engagement	Social avoidance	−0.106	[−0.178, −0.034]	0.005	0.005
	Female (vs male)	−0.022	[−0.166, 0.123]	0.761	−
	Grade 4 (vs Grade 3)	0.018	[−0.191, 0.227]	0.861	—
	Grade 5 (vs Grade 3)	−0.085	[−0.256, 0.086]	0.317	—
	School-based sport (yes vs. no)	0.288	[0.171, 0.406]	<0.001	—
	Out-of-school sport (yes vs. no)	0.418	[0.286, 0.550]	<0.001	—
BMI-excluded physical fitness	Sport engagement	0.189	[0.118, 0.260]	<0.001	<0.001
	Female (vs male)	0.020	[−0.112, 0.152]	0.759	—
	Grade 4 (vs Grade 3)	0.678	[0.373, 0.984]	<0.001	—
	Grade 5 (vs Grade 3)	−0.394	[−0.706, −0.082]	0.015	—
	School-based sport (yes vs. no)	0.221	[0.096, 0.346]	0.001	—
	Out-of-school sport (yes vs. no)	0.118	[0.008, 0.228]	0.036	—
BMI	Sport engagement	−0.141	[−0.212, −0.070]	<0.001	0.001
	Female (vs male)	−0.430	[−0.576, −0.284]	<0.001	—
	Grade 4 (vs Grade 3)	−0.008	[−0.159, 0.142]	0.910	—
	Grade 5 (vs Grade 3)	0.361	[0.135, 0.586]	0.003	—
	School-based sport (yes vs. no)	0.036	[−0.091, 0.164]	0.564	—
	Out-of-school sport (yes vs. no)	0.115	[−0.010, 0.240]	0.070	—
Overweight/obesity	Sport engagement	0.721	[0.628, 0.827]	<0.001	<0.001
	Female (vs male)	0.660	[0.484, 0.900]	0.010	—
	Grade 4 (vs Grade 3)	0.806	[0.572, 1.135]	0.208	—
	Grade 5 (vs Grade 3)	0.812	[0.510, 1.293]	0.367	—
	School-based sport (yes vs. no)	1.006	[0.723, 1.400]	0.970	—
	Out-of-school sport (yes vs. no)	1.668	[1.192, 2.334]	0.004	—

*n* = 913 students nested within 29 classrooms. Continuous outcomes and continuous focal predictors were standardized. Estimates are standardized regression coefficients (*β*) for continuous outcomes and odds ratios (ORs) for overweight/obesity. All models adjusted for sex, grade, school-based sport participation, and out-of-school sport participation. Boys, Grade 3, no school-based sport participation, and no out-of-school sport participation were the reference categories. Confidence intervals and p values were calculated using classroom-clustered CR1 standard errors with a finite-sample correction and at (28) reference distribution. Adjusted *R*^2^ values were 0.082 for sport engagement, 0.248 for BMI-excluded physical fitness, and 0.088 for BMI; Nagelkerke *R*^2^ was 0.048 for overweight/obesity. Holm-adjusted *p* values apply only to the four prespecified focal associations.

### 3.4 Associations of sport engagement with physical health indicators

After adjustment for sex, grade, school-based sport participation, and out-of-school sport participation, the total sport engagement score was positively associated with BMI-excluded physical fitness (*β* = 0.189, 95% CI [0.118, 0.260], *p* < 0.001; Holm-adjusted *p* < 0.001). The adjusted *R*^2^ for this model was 0.248.

The total sport engagement score was negatively associated with BMI (*β* = −0.141, 95% CI [−0.212, −0.070], *p* < 0.001; Holm-adjusted *p* = 0.001). The adjusted *R*^2^ for the BMI model was 0.088.

Among the 913 students included in the adjusted models, 195 (21.4%) were classified as overweight or obese. Each one-standard-deviation increase in the total sport engagement score was associated with lower odds of overweight/obesity (OR = 0.721, 95% CI [0.628, 0.827], *p* < 0.001; Holm-adjusted *p* < 0.001). Full coefficients for the covariates are reported in Table 2.

Secondary analyses of the individual physical fitness components are reported in Supplementary Table S9. After Holm correction, the total sport engagement score was positively associated with the 50-m run score (*β* = 0.194, adjusted *p* < 0.001), 1-min rope-skipping score (*β* = 0.076, adjusted *p* = 0.012), 1-min sit-up score (*β* = 0.161, adjusted *p* = 0.002), and, among Grade 5 students, the 50-m × 8 shuttle-run score (*β* = 0.274, adjusted *p* = 0.004). Associations with vital capacity (*β* = 0.024, adjusted *p* = 0.394) and sit-and-reach (*β* = 0.058, adjusted *p* = 0.113) were not statistically significant.

### 3.5 Nonlinearity and exploratory latent profile analysis

Quadratic-term results are presented in Table 3. Before multiplicity correction, positive quadratic terms were observed for the association between social avoidance and the total sport engagement score (*B* = 0.056, 95% CI [0.009, 0.103], *p* = 0.020) and for the association between the total sport engagement score and BMI-excluded physical fitness (*B* = 0.061, 95% CI [0.013, 0.108], *p* = 0.014). Neither quadratic term remained statistically significant after Holm correction (adjusted *p* = 0.061 and 0.055, respectively). The quadratic terms for BMI and overweight/obesity were also nonsignificant (adjusted *p* = 0.723 for both). Thus, none of the four prespecified associations showed statistically significant evidence of nonlinearity after multiplicity correction.

**Table 3:** Tests of potential nonlinear associations.

Outcome	Quadratic term	Metric	Estimate	95% CI	*p*	Holm-adjusted *p*
Sport engagement	Social avoidance squared	*B*	0.056	[0.009, 0.103]	0.020	0.061
BMI-excluded physical fitness	Sport engagement squared	*B*	0.061	[0.013, 0.108]	0.014	0.055
BMI	Sport engagement squared	*B*	−0.018	[−0.066, 0.030]	0.451	0.723
Overweight/obesity	Sport engagement squared	OR	0.940	[0.819, 1.078]	0.361	0.723

*n* = 913. Continuous focal predictors and continuous outcomes were standardized before the quadratic terms were formed. Models adjusted for sex, grade, school-based sport participation, and out-of-school sport participation and used the same classroom-clustered CR1 inference as Table 2. Holm adjustment was applied across the four prespecified quadratic tests. B = regression coefficient; OR = odds ratio; CI = confidence interval.

Variance inflation factors ranged from 1.02 to 1.24, no observation had a Cook’s distance greater than 1, and the logistic model converged normally. Residual inspection indicated some skewness and nonconstant variance for the continuous outcomes, supporting the use of classroom-clustered robust standard errors.

An exploratory two-class model allowing class-specific within-class variances did not terminate normally and produced a boundary solution, with one class having indicator means of 4.00 and variances approaching zero. This model was therefore not retained. The equal-variance models with two through six classes terminated normally, and the best log-likelihood was replicated for every model. AIC, BIC, and adjusted BIC continued to decrease through the six-class solution, whereas entropy ranged from 0.791 to 0.851. The VLMR-LRT and adjusted LMR-LRT were statistically significant through the four-class solution but did not support the five-class model over the four-class model or the six-class model over the five-class model. In contrast, the BLRT remained statistically significant for all comparisons. The enumeration criteria therefore did not converge on an unambiguous optimal solution. Complete results are reported in Supplementary Tables S5–S8.

The four-class solution continued to represent largely ordered increases across the three indicators, whereas the five- and six-class solutions introduced smaller classes and some dimension-specific deviations. For descriptive purposes, the three-class solution was therefore used as a parsimonious representation of low (11.0%), moderate (39.7%), and high (49.3%) engagement levels rather than as a uniquely optimal model. The cognitive, affective, and relational indicator means increased broadly in parallel across the three classes, and the corresponding average posterior probabilities were 0.937, 0.905, and 0.930. These findings suggest that the exploratory classes primarily represented ordered differences in overall engagement rather than stable, qualitatively distinct configurations. Most likely class membership was not used in the outcome analyses.

## 4 Discussion

This study examined the associations among social avoidance, sport engagement, and physical health indicators in children in Grades 3–5. Higher social avoidance was associated with lower total sport engagement, whereas higher sport engagement was associated with better BMI-excluded physical fitness, lower BMI, and lower concurrent odds of overweight/obesity. These associations remained statistically significant after adjustment for the prespecified covariates and correction for multiple testing, although their magnitudes were small to modest. None of the quadratic terms remained statistically significant after correction. The exploratory LPA further indicated that cognitive, affective, and relational engagement varied mainly along a common continuum rather than forming clearly distinct configurations. Accordingly, the principal findings concern continuous, cross-sectional associations and should not be interpreted as evidence of causal relationships or discrete engagement-based risk groups.

### 4.1 Social avoidance and sport engagement

Higher social avoidance was associated with lower total sport engagement after adjustment for sex, grade, school-based sport participation, and out-of-school sport participation. This finding indicates that social avoidance and lower psychological engagement in sport co-occurred in the present sample. However, the magnitude of the association was small, and its direction cannot be determined from the cross-sectional data.

The sport engagement measure assessed children’s psychological engagement in sport generally rather than in a single setting. The present study therefore cannot determine whether the association with social avoidance was stronger in physical education, other school-based sport activities, or out-of-school sport. Moreover, school-based and out-of-school sport participation were measured as binary indicators. Adjustment for these variables accounted for reported participation status but not participation frequency, duration, intensity, or the quality of children’s sport experiences.

Children’s sport participation occurs within broader support systems involving schools, community organizations, parents, and coaches (51, 52). Developmental models also characterize childhood as a period of varied and exploratory sport participation (53, 54). Interest development research further suggests that children’s involvement may develop from situational interest to more stable individual interest (55, 56). These perspectives provide a developmental context for understanding sport engagement, but the present study did not assess changes in interest, participation history, or adult support.

Sport participation may also include interpersonal and evaluative experiences. Physical education has been linked to students’ personal and social development (57), while physical performance may be related to peer interaction and collaboration (58). In Beijing, physical education and health assessments may increase the attention given to fitness testing and sport performance by schools and families (59, 60). Previous studies have also linked physical self-perceptions with continued participation in physical education (61), physical performance with peer relations and evaluation (62), and shyness with children’s experiences in organized sport settings (63). Taken together, this literature suggests that perceived competence, peer climate, and performance-related experiences may be relevant to the association between social avoidance and sport engagement. However, these variables were not measured in the present study. Their roles should therefore be treated as hypotheses for future research rather than mechanisms demonstrated by the current findings.

The quadratic association between social avoidance and sport engagement did not remain statistically significant after correction for multiple testing. The findings therefore do not support the previous interpretation that socially avoidant children were concentrated within a distinctive moderate-engagement group. Nor do they provide robust evidence of a threshold at which the association becomes stronger. Within the present sample, the relationship is more appropriately described as a small overall association across the engagement continuum.

### 4.2 Sport engagement and physical health indicators

Higher sport engagement was associated with better BMI-excluded physical fitness, lower BMI, and lower concurrent odds of overweight/obesity. These associations were small to modest. Given the cross-sectional design, the findings indicate that sport engagement and physical health characteristics co-occurred, but they do not establish whether engagement preceded the physical health differences or whether children’s physical health characteristics were related to their engagement in sport.

The secondary component-level analyses provided a more specific interpretation of the physical fitness result. Sport engagement was positively associated with the 50-m run, 1-min rope-skipping, 1-min sit-up, and, among Grade 5 students, the 50-m × 8 shuttle-run scores. In contrast, the associations with vital capacity and sit-and-reach were not statistically significant after correction for multiple testing. The relationship between sport engagement and physical fitness was therefore not uniform across all fitness components.

Removing the BMI component from the primary fitness score eliminated the direct mathematical overlap between physical fitness and BMI. The association between sport engagement and BMI-excluded physical fitness was therefore not attributable to including BMI in both measures. However, BMI and overweight/obesity status are not independent outcomes because weight status was classified using BMI thresholds. These findings should be understood as related results within the same weight-related domain rather than as independent replications.

Previous school-based physical activity interventions have reported improvements in some components of health-related fitness (64), while studies involving athletes have examined relationships among psychological engagement, practice processes, and performance (65, 66). Previous research has also related BMI to physical activity, sedentary behavior, school context, and family factors (67, 68). In addition, youth sport research has emphasized that the outcomes associated with sport participation may vary according to the quality of the sport experience and support from parents, peers, and coaches (69).

This literature suggests that the relationships among sport engagement, fitness, and weight-related indicators may involve multiple and potentially reciprocal processes. Nevertheless, the present study did not measure physical activity exposure, practice quality, perceived competence, or social support. It cannot determine which factors contributed to the observed associations. These possible explanations should therefore be examined in longitudinal research rather than inferred from the current findings.

The quadratic terms for BMI-excluded physical fitness, BMI, and overweight/obesity did not remain statistically significant after correction for multiple testing. The present analyses therefore provide no robust evidence of thresholds, plateaus, or other nonlinear patterns. This does not demonstrate that the relationships are strictly linear in other samples. It indicates only that the current data did not provide sufficient evidence of departure from linearity within the observed range.

### 4.3 Exploratory latent structure of sport engagement

The exploratory LPA did not identify an unequivocally optimal number of classes. Some statistical criteria continued to favor additional classes, whereas other likelihood-ratio tests did not support the more complex solutions. More importantly, cognitive, affective, and relational engagement generally increased in parallel across the candidate solutions. The classes therefore appeared to subdivide a common engagement continuum rather than represent qualitatively different configurations.

The three-class solution was retained only as a parsimonious exploratory representation of low, moderate, and high engagement levels. The distributions of the engagement indicators further limit its interpretation. A substantial proportion of students selected the highest response options, particularly for affective and relational engagement. These bounded and ceiling-inflated distributions may have contributed to the separation of students at the upper end of the continuum. The former very high engagement class should therefore not be interpreted as a separate type of sport engagement.

Overall, the exploratory LPA provides a descriptive illustration that the three engagement dimensions generally varied together. It does not provide clear evidence of configurations that add substantively different information beyond the continuous total score. Accordingly, most-likely class membership was not used in the primary outcome analyses, and no distal-outcome comparisons were conducted across the exploratory classes. The current findings do not support using these classes to identify psychosocial or physical health risk groups.

### 4.4 Practical implications

Because the associations remained after adjustment for the two binary participation indicators, psychological engagement may capture information not represented by participation status alone. This group-level association does not establish that engagement assessment improves individual identification or intervention decisions.

Based on the broader literature concerning children’s social experiences and participation in physical education (57, 58, 61–64, 69), small-group practice, peer pairing, differentiated tasks, and feedback based on personal progress may be considered when supporting students who show social avoidance or limited engagement. Reducing excessive public comparison and allowing students to progress gradually from small-group practice to more visible performance may also be considered. For students with lower physical fitness or higher BMI, tiered activities and progress-based feedback may provide alternatives to an excessive emphasis on direct performance ranking.

These approaches are informed by the broader literature rather than derived directly from the present findings, and their effectiveness remains to be evaluated. The current study did not assess classroom behavior or examine the effects of these strategies on social avoidance, sport engagement, physical fitness, or weight-related indicators. They should therefore be treated as potential approaches for future evaluation rather than interventions supported by the current data.

The sport engagement measure used in this study was also not developed as an individual screening or classroom observation instrument. The findings do not provide a validated cutoff for identifying students with low engagement. Future research could develop brief, context-specific observational tools and examine their reliability, validity, and agreement with children’s self-reports before recommending their routine use.

### 4.5 Strengths, limitations, and future directions

This study has several strengths. First, the primary analyses treated sport engagement as a continuous construct, consistent with the largely parallel variation across its cognitive, affective, and relational dimensions. Second, classroom-clustered inference accounted for the nesting of students within classrooms, and a finite-sample correction was applied because the number of classrooms was modest. Third, the analyses included correction for multiple testing and examinations of potential nonlinearity. Fourth, recalculating physical fitness without its BMI component removed direct mathematical overlap with BMI. The component-level analyses also showed that the overall fitness finding did not apply equally to all fitness indicators. Finally, reporting the ceiling effects and conflicting class-enumeration criteria reduced the risk of overinterpreting the exploratory LPA.

Several limitations should be noted. First, the cross-sectional design does not permit conclusions about temporal ordering or causality. Second, the sample was drawn from one school in Beijing, limiting generalizability to other schools, regions, and educational contexts. Included and excluded students also differed slightly in social avoidance, meaning that selection bias cannot be completely excluded.

Third, social avoidance and sport engagement were assessed by child self-report. Although both measures showed acceptable psychometric properties, no formal cognitive interviews were conducted with the present sample. Sport engagement was assessed across sport contexts rather than separately for physical education, other school-based sport activities, and out-of-school sport. School-based and out-of-school sport participation was included in the adjusted models as binary variables. Although information on sport type, weekly frequency, session duration, and years of participation was also collected, these variables were not included in the primary models; activity intensity and objectively measured physical activity were unavailable. Therefore, residual confounding related to sport type and participation dose cannot be excluded.

Fourth, the study did not assess several factors discussed as possible explanations, including perceived competence, physical self-perception, teacher support, peer climate, family support, public-performance pressure, and classroom behavior. These factors cannot be treated as mechanisms underlying the observed associations. Fifth, the number of classrooms was modest. Although the finite-sample correction improved the classroom-robust inference, uncertainty related to the number of clusters remains. Sixth, the physical fitness indicators came from routine school testing, with one recorded result for each test and no research-specific standardized warm-up or repeat-trial procedure. BMI also cannot distinguish fat mass from lean mass. Finally, ceiling responses and conflicting model-selection criteria limit the interpretation of the exploratory LPA.

Future studies should use longitudinal designs to clarify the temporal relationships among social avoidance, sport engagement, and physical health indicators. Multischool samples would improve generalizability and allow classroom- and school-level variation to be examined more precisely. Setting-specific engagement measures could determine whether the observed associations differ across physical education, other school-based sport activities, and out-of-school sport. Objective physical activity measurement and direct assessment of the proposed psychological and social factors would allow potential mechanisms to be tested. Experimental studies are also needed to evaluate whether cooperative, differentiated, and lower-pressure teaching approaches can improve sport engagement and related physical health indicators.

## 5 Conclusion

In this cross-sectional study of children in Grades 3–5 from one Beijing school, higher social avoidance was associated with lower total sport engagement. Higher sport engagement was associated with better BMI-excluded physical fitness, lower BMI, and lower concurrent odds of overweight/obesity. All four focal associations remained statistically significant after Holm correction and were small to modest in magnitude, whereas none of the four prespecified quadratic terms remained statistically significant after the same correction. The exploratory LPA did not identify an unequivocally optimal class solution, and the retained three-class representation primarily reflected ordered differences along a general engagement continuum rather than qualitatively distinct configurations. Most-likely class membership was not used in the outcome analyses. The findings are therefore limited to the observed cross-sectional associations and do not establish temporal or causal relationships or support profile-based risk classification.

## Data Availability

The original contributions presented in the study are included in the article/Supplementary material, further inquiries can be directed to the corresponding author.
