# Supplementary material for: Associations of social avoidance and sport engagement with physical health indicators among schoolchildren: a cross-sectional study
Source: Front Public Health. 2026 Sep 21;14:1890401. doi: 10.3389/fpubh.2026.1890401 (PMC13635328; doi:10.3389/fpubh.2026.1890401)
Supplement: Supplementary file 1 [file Table_1.DOCX]

**Supplementary Table S1.** Participant screening and comparisons between included and excluded students

**Panel A.** Sequential participant screening

| Screening step | Excluded, n (%) | Remaining, n |
| --- | --- | --- |
| Original records | – | 1,110 |
| Missing sex or grade information | 13 (1.2) | 1,097 |
| Missing social avoidance item(s) | 20 (1.8) | 1,077 |
| Missing sport engagement item(s) | 56 (5.0) | 1,021 |
| Sport engagement response outside the permitted 1–4 range | 11 (1.0) | 1,010 |
| Missing physical fitness or BMI data | 50 (4.5) | 960 |
| Unverified classroom identifier | 37 (3.3) | 923 |
| Total excluded from the analytical sample | 187 (16.8) | 923 |
| Missing school–based or out–of–school sport participation covariates | 10 (1.1) | 913 |

Percentages for the first six exclusion steps were calculated using the original dataset (*N* = 1,110). The percentage for missing sport–participation covariates was calculated using the analytical sample (*N* = 923).

**Panel B.** Variable–level missingness and invalid responses in the original dataset

| Variable or data requirement | Missing or invalid, n | Percentage |
| --- | --- | --- |
| Sex | 12 | 1.1 |
| Grade | 2 | 0.2 |
| At least one social avoidance item | 21 | 1.9 |
| At least one sport engagement item missing | 58 | 5.2 |
| At least one sport engagement item outside the permitted 1–4 range | 12 | 1.1 |
| Sport engagement score unavailable because of missing or out–of–range item(s) | 69 | 6.2 |
| Data required to calculate the BMI–excluded physical fitness score | 95 | 8.6 |
| BMI and weight–status classification | 54 | 4.9 |
| Classroom identifier missing or unverifiable | 92 | 8.3 |
| School–based sport participation | 3 | 0.3 |
| Out–of–school sport participation | 10 | 0.9 |

**Panel C.** Comparisons between included and excluded students using available data

| Variable | Included students | Excluded students with available data | Test statistic | *p* | Effect size |
| --- | --- | --- | --- | --- | --- |
| Sex, n (%) | n = 923 | n = 175 | χ² = 0.885 | .347 | Cramér’s V = .028 |
| Boys | 478 (51.8) | 98 (56.0) | − | − | − |
| Girls | 445 (48.2) | 77 (44.0) | − | − | − |
| Grade, n (%) | n = 923 | n = 185 | χ² = 3.311 | .191 | Cramér’s V = .055 |
| Grade 3 | 372 (40.3) | 63 (34.1) | − | − | − |
| Grade 4 | 288 (31.2) | 69 (37.3) | − | − | − |
| Grade 5 | 263 (28.5) | 53 (28.6) | − | − | − |
| Social avoidance, mean ± SD | 5.61 ± 2.41 (n = 923) | 6.16 ± 3.12 (n = 166) | *t*(201.9) = −2.184 | .030 | *d* = −.220 |
| Sport engagement, mean ± SD | 27.35 ± 6.54 (n = 923) | 26.71 ± 6.79 (n = 118) | *t*(146.1) = 0.958 | .340 | *d* = .097 |
| BMI–excluded physical fitness, mean ± SD | 101.29 ± 12.10  (n = 923) | 99.12 ± 11.67  (n = 92) | *t*(111.4) = 1.691 | .094 | *d = .180* |
| BMI, kg/m², mean ± SD | 17.62 ± 3.24 (n = 923) | 17.75 ± 3.73 (n = 133) | t(161.9) = −0.390 | .697 | *d* = −.040 |

Note. Screening criteria in Panel A were applied sequentially and were therefore mutually exclusive. Variable–level missingness in Panel B was calculated separately for each variable in the original dataset (*N* = 1,110); categories may overlap because a student could have missing data on more than one variable. Comparisons in Panel C used all available data, resulting in different sample sizes among excluded students. Pearson’s chi–square tests were used for categorical variables, and Welch’s independent–samples t tests were used for continuous variables. Cohen’s d was calculated as the standardized mean difference between included and excluded students; positive values indicate higher scores among included students. BMI, body mass index.

**Supplementary Table S2.** Sex- and grade-specific BMI thresholds for weight-status classification

| Sex | Grade | Underweight | Normal weight | Overweight | Obesity |
| --- | --- | --- | --- | --- | --- |
| Boys | Grade 3 | ≤ 13.8 | 13.9 – 19.4 | 19.5 – 22.1 | ≥ 22.2 |
|  | Grade 4 | ≤ 14.1 | 14.2 – 20.1 | 20.2 – 22.6 | ≥ 22.7 |
|  | Grade 5 | ≤ 14.3 | 14.4 – 21.4 | 21.5 – 24.1 | ≥ 24.2 |
| Girls | Grade 3 | ≤ 13.5 | 13.6 – 18.6 | 18.7 – 21.1 | ≥ 21.2 |
|  | Grade 4 | ≤ 13.6 | 13.7 – 19.4 | 19.5 – 22.0 | ≥ 22.1 |
|  | Grade 5 | ≤ 13.7 | 13.8 – 20.5 | 20.6 – 22.9 | ≥ 23.0 |

## Note. BMI is expressed in kg/m². Weight–status thresholds were based on the sex – and grade–specific criteria of the Chinese National Student Physical Fitness Standard (2014 Revision) (43).

## **Supplementary Table S3.** Distributions and ceiling-response proportions of sport engagement scores and items

**Panel A.** Distributions and maximum–score proportions for the overall sport engagement score and subscales

| Score | Mean | SD | Median | IQR | Skewness | Kurtosis | At maximum, n (%) |
| --- | --- | --- | --- | --- | --- | --- | --- |
| Overall sport engagement | 27.35 | 6.54 | 28.00 | 23.00–33.00 | −0.641 | −0.164 | 105 (11.4) |
| Cognitive engagement | 3.047 | .777 | 3.000 | 2.667 – 3.667 | –.622 | –.257 | 191 (20.7) |
| Affective engagement | 3.073 | .802 | 3.000 | 2.667 – 3.667 | –.668 | –.245 | 224 (24.3) |
| Relational engagement | 2.996 | .852 | 3.000 | 2.333 – 3.667 | –.600 | –.491 | 214 (23.2) |

**Panel B.** Item–level distributions and maximum–response proportions

| Item | Mean | SD | Skewness | Kurtosis | Response = 4, n (%) |
| --- | --- | --- | --- | --- | --- |
| Cognitive item 1 | 3.426 | .810 | –1.400 | 1.342 | 544 (58.9) |
| Cognitive item 2 | 3.025 | .957 | –.563 | –.778 | 365 (39.5) |
| Cognitive item 3 | 2.689 | 1.080 | –.212 | –1.240 | 274 (29.7) |
| Affective item 1 | 3.310 | .910 | –1.144 | .288 | 512 (55.5) |
| Affective item 2 | 3.064 | .984 | –.703 | –.636 | 394 (42.7) |
| Affective item 3 | 2.844 | 1.119 | –.440 | –1.207 | 355 (38.5) |
| Relational item 1 | 3.222 | .950 | –.996 | –.086 | 470 (50.9) |
| Relational item 2 | 2.932 | 1.043 | –.525 | –.970 | 357 (38.7) |
| Relational item 3 | 2.834 | 1.087 | –.439 | –1.123 | 329 (35.6) |

Note. N = 923. The overall sport engagement score was calculated as the sum of nine items and ranged from 9 to 36. Each subscale score was calculated as the mean of three items and ranged from 1 to 4. “At maximum” indicates an overall score of 36 or a subscale mean of 4.00. A total of 144 students (15.6%) obtained the maximum score on both the affective and relational engagement subscales. At the item level, maximum responses indicate a response of 4 (“often”). IQR = interquartile range; SD = standard deviation.

**Supplementary Table S4.** Classroom intraclass correlations and design effects

| Variable | ICC | Design effect | Grade-adjusted residual ICC |
| --- | --- | --- | --- |
| Social avoidance | .011 | 1.35 | .011 |
| Overall sport engagement | .023 | 1.72 | .023 |
| Cognitive engagement | .023 | 1.71 | .026 |
| Affective engagement | .016 | 1.50 | .014 |
| Relational engagement | .020 | 1.63 | .016 |
| BMI-excluded physical fitness | .289 | 9.90 | .101 |
| BMI | .050 | 2.53 | .019 |

Note. N = 923 students nested within 29 classrooms. Mean classroom size was 31.83 students (range = 9–41). ICCs were estimated using random-intercept models. Grade-adjusted residual ICCs were obtained from models including grade as a fixed effect. Design effects were calculated from the unadjusted ICCs as 1 + (mean classroom size − 1) × ICC. ICC = intraclass correlation coefficient.

**Supplementary Table S5.** Fit indices and diagnostics for equal-variance latent profile models with two through six classes

**Panel A.** Fit indices, class sizes, and estimation diagnostics from classroom-complex models

| K | Free parameters | LL | AIC | BIC | aBIC | Entropy | Smallest estimated class, % | | Smallest modal n (%) | Best LL replicated | Status |
| --- | --- | --- | --- | --- | --- | --- | --- | --- | --- | --- | --- |
| 2 | 10 | –2799.788 | 5619.577 | 5667.853 | 5636.094 | .834 | 33.1 | 290 (31.4) | | Yes | Normal |
| 3 | 14 | –2604.866 | 5237.731 | 5305.318 | 5260.856 | .830 | 11.0 | 96 (10.4) | | Yes | Normal |
| 4 | 18 | –2538.262 | 5112.524 | 5199.422 | 5142.256 | .791 | 7.3 | 65 (7.0) | | Yes | Normal |
| 5 | 22 | –2511.146 | 5066.291 | 5172.499 | 5102.630 | .851 | 5.5 | 51 (5.5) | | Yes | Normal |
| 6 | 26 | –2464.543 | 4981.087 | 5106.605 | 5024.032 | .826 | 5.7 | 53 (5.7) | | Yes | Normal |

**Panel B.** Likelihood-ratio tests from non-complex enumeration models

| Classes, *k* | VLMR-LRT, *p* | Adjusted LMR-LRT, *p* | BLRT, *p* |
| --- | --- | --- | --- |
| 2 | < .001 | < .001 | < .001 |
| 3 | .0002 | .0003 | < .001 |
| 4 | .0051 | .0059 | < .001 |
| 5 | .0850 | .0906 | < .001 |
| 6 | .1437 | .1511 | < .001 |

Note. N = 923. Panel A presents results from classroom-complex models estimated using robust maximum likelihood and TYPE = MIXTURE COMPLEX, with 29 classrooms specified as clusters. Within-class indicator variances were constrained to equality across classes, and within-class covariances were fixed at zero. The two-class model used 1,000 initial-stage random starts and 200 final-stage optimizations; the three- through six-class models used 2,000 initial-stage starts and 400 final-stage optimizations. All models used 100 initial-stage iterations. The best log-likelihood was replicated, and all models terminated normally. Panel B presents VLMR-LRT, adjusted LMR-LRT, and BLRT results from otherwise equivalent non-complex enumeration models because these tests were not available with the classroom-complex specification. Each test compares the k-class model with the k − 1 class model. All 500 requested bootstrap draws were completed successfully. AIC = Akaike information criterion; aBIC = sample-size-adjusted Bayesian information criterion; BIC = Bayesian information criterion; BLRT = bootstrapped likelihood-ratio test; LL = log-likelihood; LMR-LRT = Lo–Mendell–Rubin adjusted likelihood-ratio test; VLMR-LRT = Vuong – Lo – Mendell – Rubin likelihood-ratio test.

**Supplementary Table S6.** Class proportions, indicator means, and average posterior probabilities for the exploratory three-class solution

| Descriptive label | Estimated class, % | Modal n (%) | Cognitive mean | Affective mean | Relational mean | Average posterior probability |
| --- | --- | --- | --- | --- | --- | --- |
| Low engagement | 11.0 | 96 (10.4) | 1.750 | 1.627 | 1.562 | .937 |
| Moderate engagement | 39.7 | 362 (39.2) | 2.717 | 2.750 | 2.644 | .905 |
| High engagement | 49.3 | 465 (50.4) | 3.603 | 3.657 | 3.601 | .930 |

Note. N = 923. Estimated class percentages are model-based estimates, whereas modal n (%) values are based on most likely class membership. Indicator means are reported on the original 1–4 scale. Average posterior probabilities are the diagonal classification probabilities for students assigned to each class. The labels describe ordered levels of sport engagement and should not be interpreted as qualitatively distinct psychological types.

**Supplementary Table S7.** Posterior classification diagnostics for the exploratory three-class latent profile solution

**Panel A.** Posterior latent-class probabilities conditional on most likely class assignment

| Most likely class (row) | Low latent class | Moderate latent class | High latent class |
| --- | --- | --- | --- |
| Low engagement | .937 | .063 | .000 |
| Moderate engagement | .033 | .905 | .062 |
| High engagement | .000 | .070 | .930 |

**Panel B.** Most likely class-assignment probabilities conditional on latent-class membership

| Latent class (row) | Low most likely class | Moderate most likely class | High most likely class |
| --- | --- | --- | --- |
| Low latent class | .882 | .118 | .000 |
| Moderate latent class | .017 | .894 | .089 |
| High latent class | .000 | .049 | .951 |

Note. N = 923. Rows sum to 1.00, subject to rounding. Diagonal values represent correct classification probabilities. Entropy for the three-class solution was .830. Most likely class membership was not treated as an observed predictor or grouping variable in the revised primary outcome analyses. Associations with social avoidance and health-related outcomes were examined using the overall sport engagement score.

**Supplementary Table S8.** Indicator means and estimated class proportions for latent profile solutions with three through six classes

| Number of classes | Reordered class | Estimated class, % | Cognitive mean | Affective mean | Relational mean |
| --- | --- | --- | --- | --- | --- |
| 3 | 1 | 11.0 | 1.750 | 1.627 | 1.562 |
| 3 | 2 | 39.7 | 2.717 | 2.750 | 2.644 |
| 3 | 3 | 49.3 | 3.603 | 3.657 | 3.601 |
| 4 | 1 | 7.3 | 1.561 | 1.395 | 1.456 |
| 4 | 2 | 22.0 | 2.418 | 2.413 | 2.255 |
| 4 | 3 | 35.2 | 3.062 | 3.090 | 3.054 |
| 4 | 4 | 35.5 | 3.726 | 3.808 | 3.713 |
| 5 | 1 | 5.5 | 1.508 | 1.211 | 1.485 |
| 5 | 2 | 10.7 | 2.393 | 1.983 | 2.111 |
| 5 | 3 | 20.4 | 2.552 | 2.650 | 2.543 |
| 5 | 4 | 28.4 | 3.192 | 3.140 | 3.156 |
| 5 | 5 | 35.0 | 3.659 | 3.889 | 3.638 |
| 6 | 1 | 5.7 | 1.523 | 1.218 | 1.496 |
| 6 | 2 | 11.8 | 2.423 | 2.020 | 2.160 |
| 6 | 3 | 17.4 | 2.468 | 2.696 | 2.449 |
| 6 | 4 | 17.4 | 3.281 | 2.910 | 3.279 |
| 6 | 5 | 17.6 | 3.123 | 3.451 | 3.039 |
| 6 | 6 | 30.1 | 3.732 | 3.925 | 3.733 |

Note. N = 923. Class proportions are model-estimated percentages rather than proportions based on most likely class assignment. Within each solution, classes were reordered from lower to higher engagement according to the unweighted mean of the three class-specific indicator means. The reordered-to-original Mplus class mappings were 1–2–3 for the three-class solution, 3–1–4–2 for the four-class solution, 2–3–1–4–5 for the five-class solution, and 1–3–4–5–2–6 for the six-class solution. All indicators were scored from 1 to 4, with higher values indicating greater engagement.

**Supplementary Table S9.** Adjusted associations between overall sport engagement and individual physical fitness component scores

| Physical fitness component | n | Mean | SD | Standardized *β* | 95% CI | *p* | Holm-adjusted *p* |
| --- | --- | --- | --- | --- | --- | --- | --- |
| Vital capacity | 913 | 89.80 | 10.94 | .024 | [−.033, .081] | .394 | .394 |
| 50-m run | 913 | 77.06 | 16.01 | .194 | [.120, .269] | < .001 | < .001 |
| Sit-and-reach | 913 | 86.44 | 13.54 | .058 | [−.002, .118] | .056 | .113 |
| 1-min rope skipping | 913 | 99.25 | 3.56 | .076 | [.026, .125] | .004 | .012 |
| 1-min sit-ups | 913 | 80.21 | 11.97 | .161 | [.081, .241] | < .001 | .002 |
| 50-m × 8 shuttle run, Grade 5 | 255 | 71.68 | 17.21 | .274 | [.145, .404] | .001 | .004 |

Note. Component scores are standardized scores derived from the Chinese National Student Physical Fitness Standard. Continuous variables were standardized before analysis. Models used classroom-clustered CR1 standard errors and adjusted for sex, grade, school-based sport participation, and out-of-school sport participation. The Grade 5 shuttle-run model adjusted for sex and the two sport-participation variables because it was restricted to Grade 5 students. Holm correction was applied across the six component analyses. CI = confidence interval; SD = standard deviation.
